# Nanosecond Intersystem Crossing Times in Fullerene Acceptors: Implications for Organic Photovoltaic Diodes

**DOI:** 10.1002/adma.201400846

**Published:** 2014-06-06

**Authors:** Philip C Y Chow, Sebastian Albert-Seifried, Simon Gélinas, Richard H Friend

**Affiliations:** Cavendish Laboratory, University of CambridgeCB3 0HE, United Kingdom

**Keywords:** organic electronics, solar cells, photovoltaic devices, fullerenes, electronic processes

Functionalized fullerene derivatives are frequently used in solution-processed organic photovoltaic cells (OPVs), with power conversion efficiencies now exceeding 10%.[1] Although substantial improvements to device efficiencies have been achieved through developing new donor polymers (typically low-bandgap, E_g_∼1.6–1.8 eV), [6,6]-phenyl-C_61/71_-butyric acid methyl esters (PCBM) have remained one of the best electron acceptors since their introduction almost two decades ago.[2] In pristine fullerenes very high yields of triplet excitons are formed through efficient intersystem crossing (ISC) of photoexcited singlet excitons driven by the large spin-orbit interactions in the fullerene cage and the small exchange energies (∼0.2 eV).[3]–[6] We report here the ISC times for PCBM, and find that though fast (lower bounds are 0.7 ns for PC_70_BM and 1.4 ns for PC_60_BM), these times are longer than the characteristic times for dissociation of photoexcitations in OPVs. This is very important for PV operation, since the generation of triplet excitons on the PCBM domains is expected to have a detrimental effect on device performance. Besides limiting the device lifetime through reacting with oxygen to produce highly reactive radical anion, O_2_^−^,[7],[8] the PCBM triplets can transfer into low-energy triplets on the donor polymer rather than separating into charge-carriers.[9] This is very likely because the PCBM triplet energies (1.5 eV)[10] are higher than those of the polymers (typically 0.9–1.2 eV).[11] Once formed, the polymer triplets are rapidly quenched to the ground state through triplet-charge annihilation, and thus act as a very substantial loss mechanism.[12]–[14] A schematic of this mechanism and the chemical structures of PCBMs are depicted in **Figure 1**.

**Figure 1:**
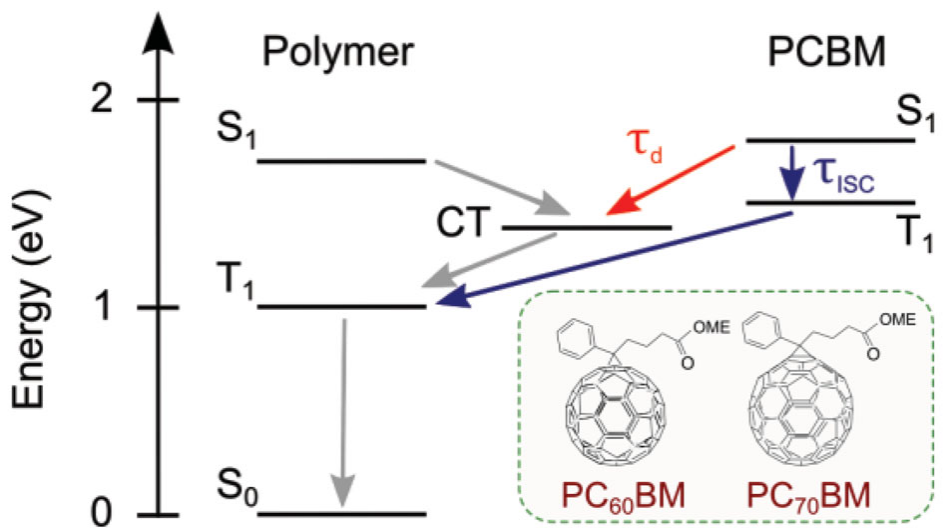
Jablonski diagram illustrating the competition between charge generation (with time constant τ_d_) and intersystem crossing (with time constant τ_ISC_) of PCBM singlet excitons (S_1_). The energies are relative to the ground state (S_0_) and all arrows describe population transfer. The formation of PCBM triplet excitons (T_1_) is detrimental to the device performance. Driven by the large energy difference, they can transfer into low-energy triplet excitons on the donor polymer, which are then quickly quenched by the charges. This loss pathway competes with charge generation, particularly in systems with high charge-transfer (CT) state energies. Note that the CT states with either spin-singlet or spin-triplet character have very similar energies due to the weak wavefunction overlap, and are therefore neglected here for simplicity. The triplet excitons can also limit the device lifetime through reacting with oxygen to form highly reactive superoxide radical anion, O_2_^−^. The molecular structures of the systems studied are shown as insets.

The light-absorbing layer of an OPV cell is a finely intermixed thin-film of the donor polymer and fullerene acceptor. The energetic offset at the interface provides the necessary driving force to overcome the strong binding energies (typically ∼ 0.3 eV) of singlet excitons resulting from the low dielectric constants of organic semiconductors (ε ∼ 3).[15] These neutral excitations are photogenerated on both the donor polymer and the fullerene domains, and charge generation occurs across the interface. For triplet excitons, the energetic offset required for dissociation is several hundred milli-electronvolts greater than for singlet excitons due to the exchange energy.[15] This process is therefore not energetically favourable in OPV systems with high charge-transfer (CT) state energies (as required to maximise open-circuit voltage).[16] It is now considered that the superior device performance achieved with fullerene derivatives such as PCBM as the acceptor is due to their ability to grow pure aggregates (about 10 nm) near finely intermixed domains.[17]–[21] The pure fullerene aggregates are considered to generate the necessary delocalised states that can sustain long-range charge separation.[22]

Here we report the ISC timescale in PCBMs (τ_ISC_), and compare them with the timescale at which charges are generated from the dissociation of photogenerated singlet excitons (τ_d_). These are not straightforward to measure because spectral features in transient optical measurements from singlet and triplet states are difficult to observe and to separate. We investigated the exciton dynamics in PC_60_BM and PC_70_BM samples using time-resolved photoluminescence (PL) spectroscopy and transient absorption spectroscopy. These samples consisted of dilute solutions (1 mg/ml in various solvents) and thin spin-coated films (∼120 nm thick on quartz substrates). In solution phase, the excitons are localised on a single fullerene and most optical transitions in the visible spectral region are forbidden due to the high structural symmetry.[23],[24] Alternatively, the intermolecular interactions in films allow certain optical transitions in the visible including the lowest-energy singlet exciton (S_1_) state. The ground state absorption and the steady-state PL spectra are shown in **Figure 2**a. The excited states in these systems are weakly emissive; we measured quantum yields of 0.20% and 0.14% for PC_60_BM and PC_70_BM solutions, respectively, and these were below the detection limit for films. For transient absorption spectroscopy, we used home-built non-collinear optical parametric amplifiers to generate broadband probe pulses with an overall spectral range spanning from 520–1600 nm.[25] As we show below, this broad spectral range is necessary to distinguish the singlet and triplet absorption features. The samples were photoexcited at between 470–530 nm throughout this work, which corresponds to a spectral region where PCBM actively absorb and contribute to the photocurrent in OPVs.[26]

**Figure 2:**
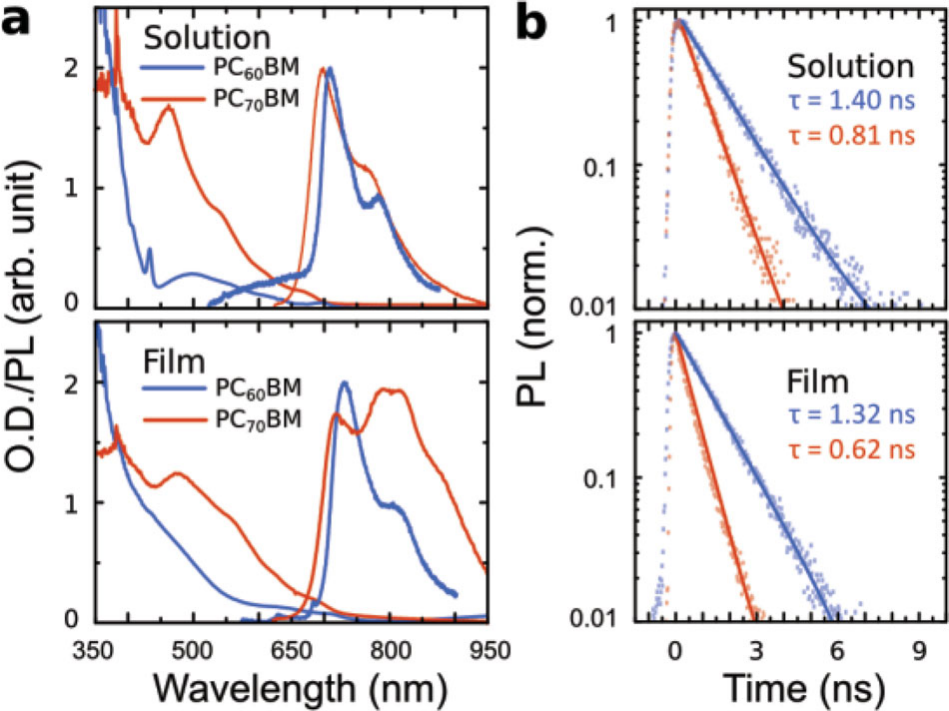
(a) Ground state absorption and photoluminescence (PL) spectra of PC_60_BM (blue) and PC_70_BM (red) of 1 mg/ml chlorobenzene solutions and spin-coated films. The PL spectra have been corrected for detector response. (b) Time evolution of PL measured at their peaks using time-correlated single photon counting with excitation wavelength of 470 nm. The PL kinetics were not dependent on the probe wavelength, which suggests that the PL spectra shown in panel (a) corresponded to the same optical transition. The solid lines show the fits of a mono-exponential decay model with the displayed time constants.

Figure 2b shows the time evolution of the PL measured at their peaks. The kinetics were fitted to a mono-exponential decay model with the displayed time constants. The good fits to the model indicate that the PL were resulted from a single excited species, which is most likely the photo-excited singlet excitons based on the short lifetimes. The PL kinetics were not dependent on the probe wavelength, which suggests that the observed PL features corresponded to the same optical transition within the accessible spectral range. We note that a slight reduction to the singlet exciton lifetimes (by ∼100 ps) was observed for solutions with strongly chlorinated solvents (Figure S1). Given that non-radiative decay should not be affected by the choice of solvent, we consider that this is due to faster ISC resulted from the increased spin-orbit interactions. The singlet lifetimes were reduced in films, which is expected because the intermolecular interaction should give rise to non-radiative decay channels. However, the small differences indicate that the dynamics of the singlets are not significantly affected, particularly in PC_60_BM.

We now turn to the transient absorption results for solutions (**Figure 3**). The negative differential transmission signals observed throughout the spectral range correspond to excited state absorptions. The transient spectra exhibited significant spectral changes as the time delay increased from picoseconds to nanoseconds (panel (a) and (d)). By employing a spectral extraction tool based on a genetic algorithm, we show that a linear combination of two distinct species, shown for both samples in panel (b) and (e), can fully explain the observed dynamics.[27] More details about the spectral extraction tool can be found in Supporting Information. The kinetics associated with these extracted spectra are shown in panel (c) and (f), fitted to a mono-exponential decay and growth model based on a shared time constant (see **Table 1** for values). The emerging spectrum was long-lived, with lifetimes of 40μs (PC_60_BM) and 140 μs (PC_70_BM) (Figure S2). On the basis of these results, we assign the initial excited state (present over picosecond timescales) to photoexcited singlets and the one emerging at later times to triplets.

**Table 1:** The measured lifetimes (τ) of singlet and triplet excitons for all samples studied in this work. The singlet exciton radiative decay rates (k_r_) were determined using the PL quantum yields discussed in the text. The remaining decay rates of singlet excitons were calculated using the relationship τ_Singlet_ = (k_r_ + k_nr_ + k_ISC_)^−1^, where nr and ISC denote non-radiative decay and intersystem crossing, respectively. Solutions were prepared in chlorobenzene

	Solutions		Films	
	PC_60_BM	PC_70_BM	PC_60_BM	PC_70_BM
τ_Singlet_ from TCSPC [ns]	1.40	0.81	1.32	0.62
τ_Singlet_ from TA [ns]	1.33	0.86	1.40	0.65
k_r_ [s^−1^]	1.4 × 10^6^	1.7 × 10^6^	> 2 × 10^6^	> 2 × 10^6^
k_nr_ + k_ISC_ [s^−1^]	7.1 × 10^8^	1.2 × 10^9^	7.1 × 10^8^	1.5 × 10^9^
τ_Triplet_ [μs]	40	140	–	–

**Figure 3:**
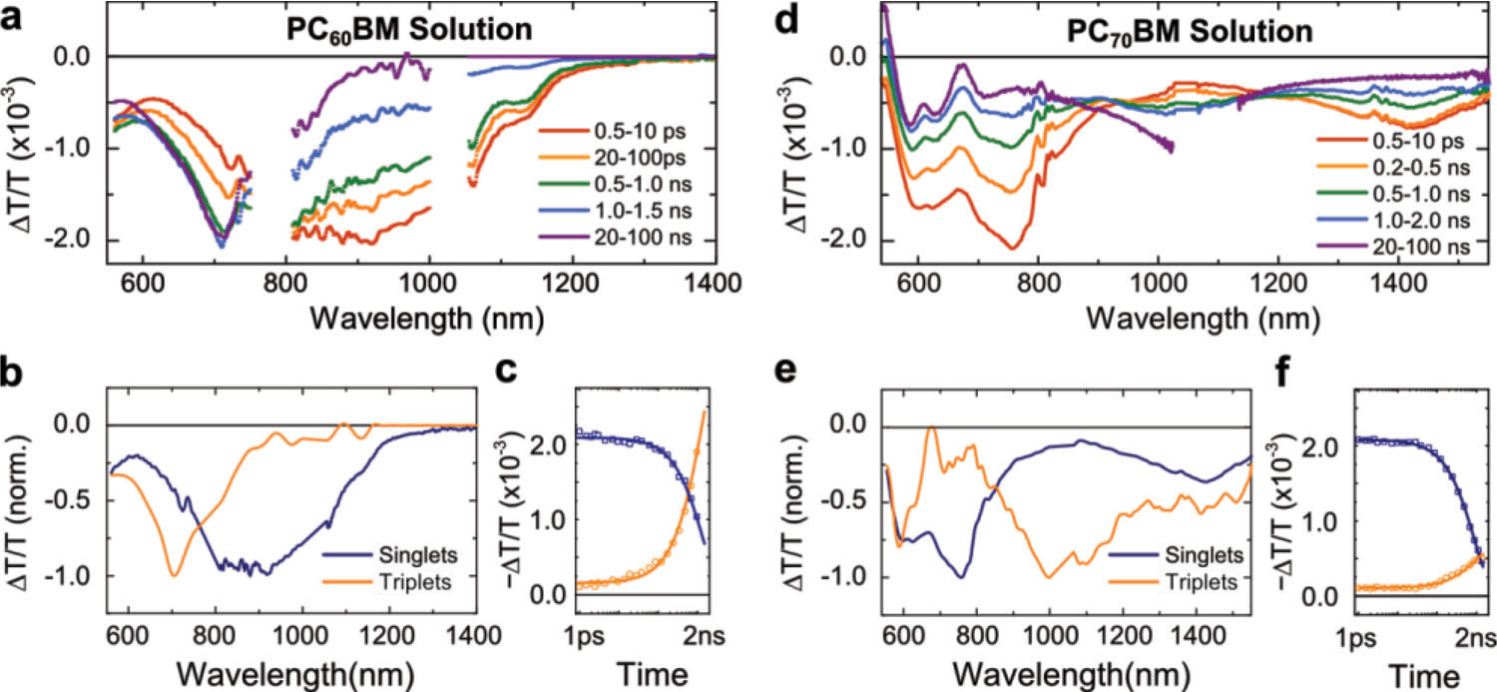
Transient absorption spectroscopy measurements of PC_60_BM (a) and PC_70_BM (d) solutions in chlorobenzene (1 mg/ml), photoexcited at 530 nm at an excitation fluence of 100μJ/cm^2^ per pulse. The lower panels show the absorption spectra (b, e) and corresponding kinetics (c, f) of the singlet and triplet excitons as extracted from the data by using a numerical method as described in the text. The singlet and triplet kinetics were fitted to a mono-exponential decay and growth model with a shared time constant. The obtained time constants are summarised in Table1.

For films, the transient spectra were much broadened compared to those observed for solutions, but exhibited similar spectral changes with time (**Figure 4**). We employed the same analysis method as for the solution measurements to deconvolute the overlapping spectral features. We show that the overall dynamics also represents a linear combination of two distinct species, which resemble the singlet and triplet absorption spectra and kinetics observed in solutions (see Table1 for values). This consistency indicates that singlet and triplet excitons are the primary excited states in films. It is clear that the exciton absorption features were broadened as a result of intermolecular interactions in these films. However, the similar kinetic lifetimes suggests that the exciton dynamics were not significantly affected.

**Figure 4:**
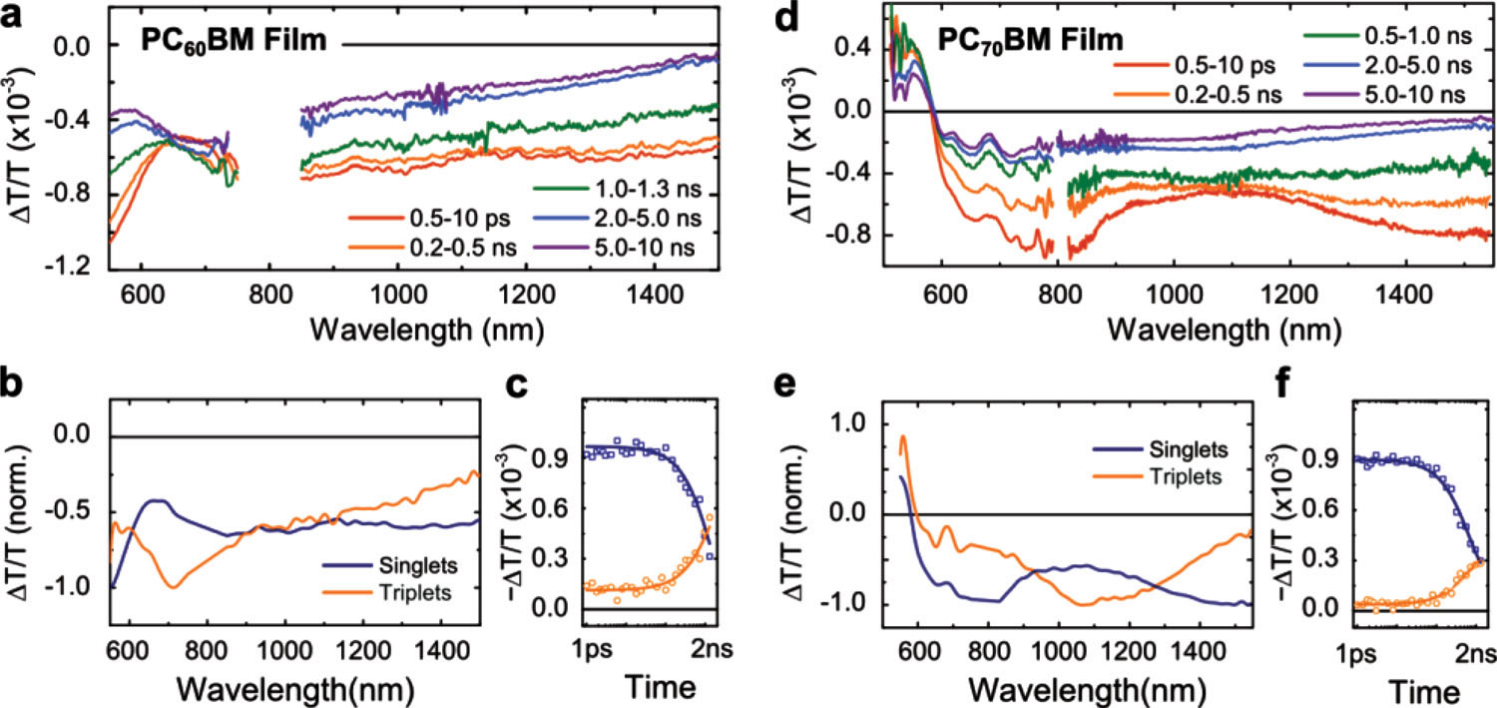
Transient absorption spectroscopy measurements of PC_60_BM (a) and PC_70_BM (d) films spin-coated on quartz substrates. The samples were photoexcited at 530 nm at an excitation fluence of 30 μJ/cm^2^ per pulse. The lower panels show the absorption spectra (b, e) and corresponding kinetics (c, f) of the singlet and triplet excitons as extracted from the data by using a numerical method as described in the text. The singlet and triplet kinetics were fitted to a mono-exponential decay and growth model with a shared time constant. The obtained time constants are summarised in Table1.

The time constants extracted from both time-resolved PL and transient absorption measurements correspond to the singlet exciton lifetime, which is given by (k_r_ + k_nr_ + k_ISC_)^−1^, where k_r_ is the radiative decay rate, k_nr_ is the non-radiative decay rate, and k_ISC_ is the ISC rate. Therefore, the extracted time constants provide a lower bound of the ISC timescales. As evident from the low PL yields, k_r_ is negligible for all samples (>10^−6^ s^−1^). Based on the high triplet yields previously observed for other fullerene derivatives in solutions, we consider that the time constants extracted for solutions to be a reasonable estimate of the ISC timescale in these systems. For films, the slight reductions in the time constants were likely resulted from increased non-radiative decay through intermolecular interactions. On the basis of these observations, we estimate an ISC timescale of ∼1.4 ns (PC_60_BM) and ∼0.7 ns (PC_70_BM) as the lower-bound values for both solutions and films. We find that these times are comparable to those previously reported for the pristine fullerene molecules. [3],[4]

The ISC timescales of PCBM singlet excitons determined here provide important implications for OPVs. As summarised in Figure 1, ISC of photoexcited singlets on the PCBM domains (k_ISC_) is a loss pathway that competes with charge generation (k_d_). In OPV blends with optimised morphologies, charge generation is typically completed at a much faster timescale.[22],[28],[29] It is therefore unlikely that the PCBM singlets generated in these blends have enough time to undergo ISC, and thus this particular loss pathway is avoided. Several recent studies have reported that recombination occurred through the triplet states of the donor polymer when blended with fullerene derivatives such as ICMA and ICBA.[13],[30] Although it has been concluded that these polymer triplets were formed via bimolecular recombination of free charges, we note that they could also be formed through ISC of the fullerene singlets if k_ISC_ > k_d_. Further investigation on the ISC timescale of these fullerene derivatives would provide valuable insight on this loss mechanism. As we have noted above, the superior device efficiencies achieved using fullerene derivatives such as PCBM is now attributed to their ability to form both finely intermixed domains and pure aggregates when blended with the polymer. While we believe that this is the primary reason, we consider that the relatively slow ISC rates of PCBMs is also a contributing factor, as it sets an upper bound to the PCBM domain sizes. This should be taken into consideration for the design of future OPV acceptors.

## Experimental Methods

*Sample Preparation*: The solutions were prepared inside a nitrogen glovebox and measured in quartz cuvettes with 1 mm transmitted path length. Films were spin-coated onto Spectrosil quartz substrates that were previously cleaned via successive sonication in acetone and isopropyl alcohol.

*Spectroscopy*: UV-visible absorption spectra were measured with a Hewlett-Packard 8453 UV−vis spectrometer. For PL measurements, the samples were photoexcited using a 470 nm pulsed laser from PicoQuant with pulse width <200 ps at fluence of 5 nJ/cm^2^. The spectra were measured with a spectrograph (SpectraPro 2500i, Princeton Instruments) coupled with a thermo-electronically cooled CCD camera (PIXIS 100-F, Princeton Instruments). A time-correlated single photon counting setup (Lifespec-ps, Edinburgh Instruments) was used to measure the PL kinetics. An integrating sphere with nitrogen flow was used for measuring the PL quantum yield. The photoexcitation pulses in transient absorption measurements were generated from two different sources. For measurements within 2ns, the excitation pulses were generated using a travelling wave optical parametric amplifier of superflourescence (TOPAS, Light Conversion) seeded with a portion of a Ti:sapphire amplifier system (Spectra-Physics Solstice) operating at 1 kHz. These pulses were delayed using a mechanical delay stage (Newport). Longer time measurements from 1ns onwards were photoexcited using the second harmonic output of a Q-switched Nd:YVO4 laser (ACE; Advanced Optical Technologies Ltd.). These pulses were delayed electronically. To cover a broad spectral range spanning from 520–1600 nm, the probe pulses were generated using home-built non-collinear optical parametric amplifiers (NOPA) each pumped by a portion of the amplifier system. The transient absorption signals measured using probe pulses from these NOPAs (each with 300—500 nm bandwidths) were then merged to provide the broad spectral window. The chirp was carefully corrected such that time zero was consistent between measurements, thereby allowing accurate spectral extraction to be performed on the ensemble. The films were measured under a dynamic vacuum (<1 × 10^−5^ mbar) during PL and transient absorption measurements.

## Supporting Information

1. TCSPC data on solutions in various solvents.

2. Triplet decay in solution, up to 100 microseconds.

3. Description of spectral extraction method.
