# Supplementary material for: Nanosecond Intersystem Crossing Times in Fullerene Acceptors: Implications for Organic Photovoltaic Diodes
Source: Adv Mater. 2014 Jun 6;26(28):4851–4. doi: 10.1002/adma.201400846 (PMC4515088; doi:10.1002/adma.201400846)
Supplement: Supplementary file 1 [file adma0026-4851-sd1.pdf]

Copyright WILEY-VCH Verlag GmbH & Co. KGaA, 69469 Weinheim, Germany, 2014.

ADVANCED MATERIALS

Supporting Information

for *Adv. Mater.*, DOI: 10.1002/adma.201400846

Nanosecond Intersystem Crossing Times in Fullerene Acceptors: Implications for Organic Photovoltaic Diodes

*Philip C. Y. Chow, Sebastian Albert-Seifried, Simon Gélinas, and Richard H. Friend**

Nanosecond Intersystem Crossing Times in Fullerene Acceptors: Implications for Organic Photovoltaic Diodes

*Philip C.Y. Chow, Sebastian Albert-Seifried, Simon Gélinas, Richard H. Friend**

Cavendish Laboratory, University of Cambridge, CB3 0HE, UK

Email: rhf10@cam.ac.uk

Supporting information 1 – TCSPC data on solutions in various solvents.

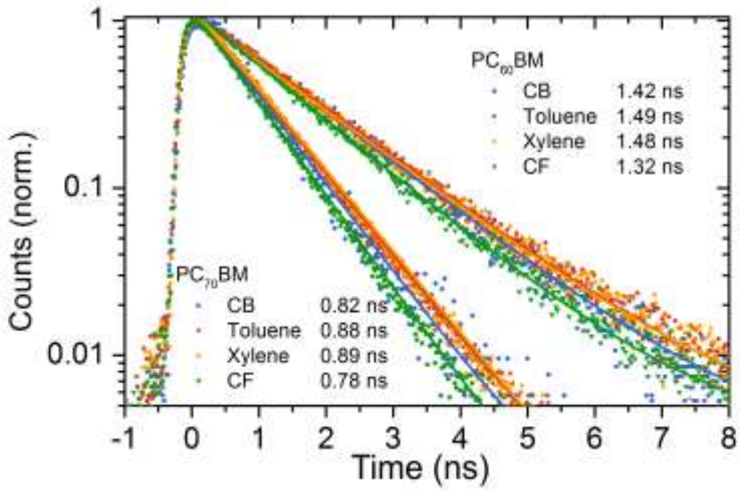

Figure S1. Time-correlated single photon counting measurements of $PC_{60}BM$ and $PC_{70}BM$ solutions prepared in different solvents (1mg/ml). The kinetics were measured at the PL maxima of ~700nm and were fitted with mono-exponential decays.

Supporting information 2 – Triplet decay in solution, up to 100 microseconds.

.

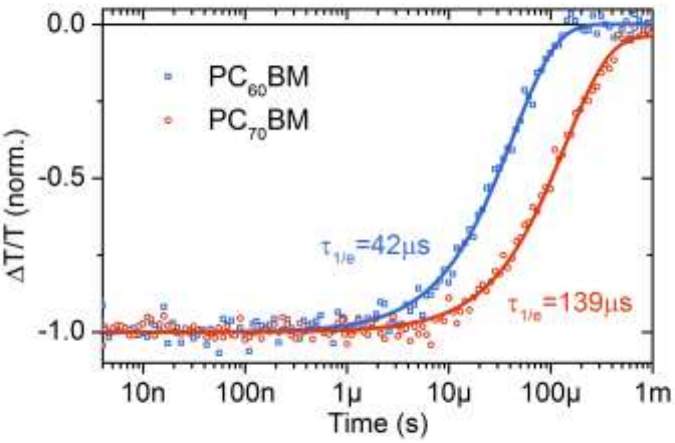

Figure S2. Transient absorption kinetics of solutions in chlorobenzene averaged over the spectral range of 600-750nm for $PC_{60}BM$ and 880-950 nm for $PC_{70}BM$. The indicated lifetimes show the values obtained by mono-exponential decay fits.

Supporting information 3 – Spectral reconstruction using a genetic algorithm

We have used the method initially presented in reference X to separate the different components that make up the transient absorption measurements. Assuming that each component has a specific spectrum whose amplitude changes over time, it is possible to represent TA data as:

$$TA(\lambda, t) = \sum_{i=1}^{n} S_i(\lambda) * A_i(t)$$

where n is the number or components, S_i is the spectrum of the ith component and A_i its time-dependent amplitude, both of which are 1D vectors whose product gives the D matrix representing the TA response of a single component. These are then summed over all components to obtain the total response.

As $TA(\lambda,t)$ is the measured data, $A_i(t)$ can be numerically reconstructed if the $S_i(\lambda)$ are known. However, this is a very large parameter space full of local minima and robust optimization techniques are required. We therefore use a genetic algorithm to find the optimal solution.

We initially generate a very large ensemble of random solutions (comprising of *n* spectra each), reconstruct their associated time-evolving amplitude (which should correspond to time-evolving populations) and calculate the fitness of each solution. To estimate the fitness, we sum the squared residual between the data and the numerical solution over all wavelengths and time points, add a penalty for non-physical solutions (e.g. negative populations), and take the inverse of that number. Solutions of higher fitness are preferably selected using a tournament method and combined to generate children, i.e. linear combination of both parent spectra, until a new generation replaces the previous one. Optimal solutions are preserved when a new generation starts (elitism) and random Gaussian mask are generated to select which sections of the parent spectra are swapped. Converged solutions are tested for reproducibility and to ensure that linear combinations of them cannot produce equally valid physical solutions.
